# Identifying fenofibrate responsive CpG sites

**DOI:** 10.1186/s12919-018-0148-3

**Published:** 2018-09-17

**Authors:** Rita Cantor, Linda Navarro, Calvin Pan

**Affiliations:** Department of Human Genetics, David Geffen School of Medicine, University of California at Los Angeles, 695 Charles E. Young Dr. South, Los Angeles, CA USA

## Abstract

As part of GAW20, we analyzed the familiality and variability of methylation to identify cytosine-phosphate-guanine (CpG) sites responsive to treatment with fenofibrate. Methylation was measured at approximately 450,000 sites in pedigree members, prior to and after 3 weeks of treatment. Initially, we aimed to identify responsive sites by analyzing the pre- and posttreatment methylation changes within individuals, but these data exhibited a confounding treatment/batch effect. We applied an alternative indirect approach by searching for CpG sites whose methylation levels exhibit a genetic response to the drug. We reasoned that these sites would exhibit highly familial and variable methylation levels posttreatment, but not pretreatment. Using a 0.1% threshold, posttreatment sibling correlation (scor) and standard deviation (SD) distributions share 16 outliers, while the corresponding pretreatment distributions share none. Comparing the pre- and posttreatment CpG outliers, 36 (8%) of SD distributions, and 449/450 (nearly 100%) of scor distributions differ. Combined, these results identify methylation sites within the *KIAA1804* and *ANAPC2* genes. Each gene also has a highly significant methylation quantitative trait locus (meQTL) (*KIAA1804: p* < 1e-200; *ANAPC2: p* < 3e-248), indicating that methylation levels at these CpG sites are driven by meQTL and fenofibrate.

## Background

Chromatin accessibility regulates gene expression. The addition of methyl groups to chromosome regions of gene initiation represses transcription, whereas loci, free from DNA methylation, allow the initiation of gene expression. The degree of epigenetic regulation at these loci varies by cell and tissue type and is responsive to genetic and environmental factors, such as treatment with a drug [1]. Recent research suggests an additional model where the degree of gene expression alters methylation levels, contributing to the notion that the relationship between methylation and gene expression is a two-way process [2]. Data available to GAW20 provide an opportunity to assess methylation levels prior to and following the administration of the lipid-lowering drug, fenofibrate. The Genetics of Lipid Lowering Drugs and Diet Network (GOLDN) study [3] includes a longitudinal study of family members who have been measured for methylation levels in blood at approximately 450,000 sites before and after 3 weeks of treatment with 160 mg/day of the lipid-lowering drug, micronized fenofibrate.

Our initial aim was to assess whether methylation levels at cytosine-phosphate-guanine (CpG) sites are responsive to fenofibrate, and then identify the most responsive. We planned to use their longitudinal differences in methylation levels in the analyses to achieve this aim; however, discussions at GAW20 highlighted confounding batch effects in methylation measures pre- and posttreatment. Adjustment was not straightforward, as none of the samples were measured in both batches, and there were no untreated individuals measured at both times to act as controls.

We remained focused on our aim, and employed an alternative indirect approach. We reasoned that there may be CpG sites where the response to fenofibrate is influenced by genetic variants. First, we know that fenofibrate is a ligand for the transcription factor, peroxisome proliferator activated receptor α, and it activates proteins that bind to transcription factor binding sites. If the genetic sequence of a site harbors a single nucleotide polymorphism (SNP), transcription levels will vary based on the allele present, which will introduce variability into the degree of gene expression, which variability may be reflected by the degree of methylation at the CpG. Such genetic effects will be reflected by increases in the heritability and variability of their methylation levels, as these are hallmarks of a genetic contribution to a quantitative trait.

In our analyses, we used familiality as a surrogate for heritability, because twin pairs are not available for analysis. We estimated familiality using the correlation of methylation levels among sibling pairs (scor), recognizing the estimate may be inflated by the effects of common environment that includes factors in addition to the treatment with fenofibrate. Variability in methylation levels will also be increased, because genetic alleles impact trait variance by making it larger. We use the standard deviation (SD) as our measure of variability. Using this approach, we identified the concordant outliers of the posttreatment methylation scor and SD distributions, and filtered the concordant outliers to identify those that were not pretreatment outliers. We interpreted these CpG sites as exhibiting a genetic response to treatment.

To generate a more complete picture of the genetic and fenofibrate influences on methylation levels at the sites identified by the outlier analysis, we assessed whether their methylation levels were influenced by methylation quantitative trait loci (meQTLs). meQTLs contribute to methylation levels directly, regardless of the treatment with fenofibrate, although it is very conceivable that the treatment could enhance their effect. There is a growing literature describing the role of meQTLs [4], although much remains to be researched and understood about their mechanisms of operation. To identify meQTLs, we tested the SNPs in the regions surrounding the fenofibrate-responsive CpG sites for significant associations with the methylation levels.

## Methods

### The study sample

Pretreatment methylation levels at approximately 450,000 sites were assessed for 995 individuals in 182 pedigrees. Pretreatment methylation level SDs were estimated in this sample. Although these individuals were pedigree members, we did not adjust SDs for family structure because each estimate was made on the same sample, and our approach ranked the estimates, but did not draw inferences that assume their independence. These estimates were used to construct the pretreatment SD distribution. Posttreatment methylation levels, assessed at 450,000 sites in 153 pedigrees containing 530 individuals, were used to construct the posttreatment SD distribution. Among the pre- and posttreatment samples, 446 individuals were common to both. Within the 182 pedigrees, there were 163 sibling pairs that had pretreatment methylation data and correlations of methylation levels in the sibling pairs were used to construct the pretreatment scor distribution. Because CpG methylation levels are not normally distributed, we used a Spearman correlation, and for consistency, the siblings in each pair were ordered by their birth order when estimating the correlations. There were 119 sibling pairs in the posttreatment sample used to construct the posttreatment scor distribution. Of all the sibling pairs, 102 were common to both samples.

### Outlier analyses

To identify the sites with the most familial and variable posttreatment methylation levels, we identified the scor and SD outliers, separately, pre- and posttreatment. Outliers were defined using an approximate 0.1% (450 sites) threshold, and identified by ranking the scor and SD CpG estimates within each distribution. R functions [5] were used to estimate pre- and posttreatment scor and SD for each of the approximately 450,000 CpG sites, generate histograms for scor and SD values, and identify their outliers and overlaps. We filtered these sites to identify concordant posttreatment scor and SD outliers that had not been pretreatment SD or scor outliers. CpG sites meeting these criteria were interpreted to exhibit a genetic pattern in their response to fenofibrate, and were termed *candidate fenofibrate-responsive CpG sites*.

### meQTL analyses

Two candidate fenofibrate-responsive genes were identified by their CpG sites, and we looked for meQTLs in their chromosome regions. Using a minor allele frequency of > 1%, and extending 1 Mb on either side of their associated CpG sites, there were 824 SNPs at *KIAA1804* and 185 at *ANAPC2* for meQTL analyses of regional SNPs and CpG methylation levels.

SNP associations were tested in fenofibrate-responsive gene regions using the *Fa*ctored *S*pectrally *T*ransformed *L*inear *M*ixed *M*odels (FaST-LMM) variance component approach, which can be used for association testing in pedigrees [6]. This software models a vector of pedigree member trait value deviations from the pedigree mean and a covariance matrix of kinship coefficients among the pedigree members. The relationships among the individuals in the study sample do not need to be specified explicitly to account for their nonindependence, as carefully chosen genome-wide association study (GWAS) SNPs genotyped on the study sample are used to estimate genetic similarity. This estimation is done using SNPs from all chromosomes except the single chromosome containing the locus being tested for association. Linear mixed models capture these relationships and a transformation of the estimated matrix of pairwise relationships speeds the analysis.

Figures illustrating the location of the associated SNPs in relation to their target gene and methylation site were generated using the LocusZoom software [7].

## Results

**Fig. 1:**
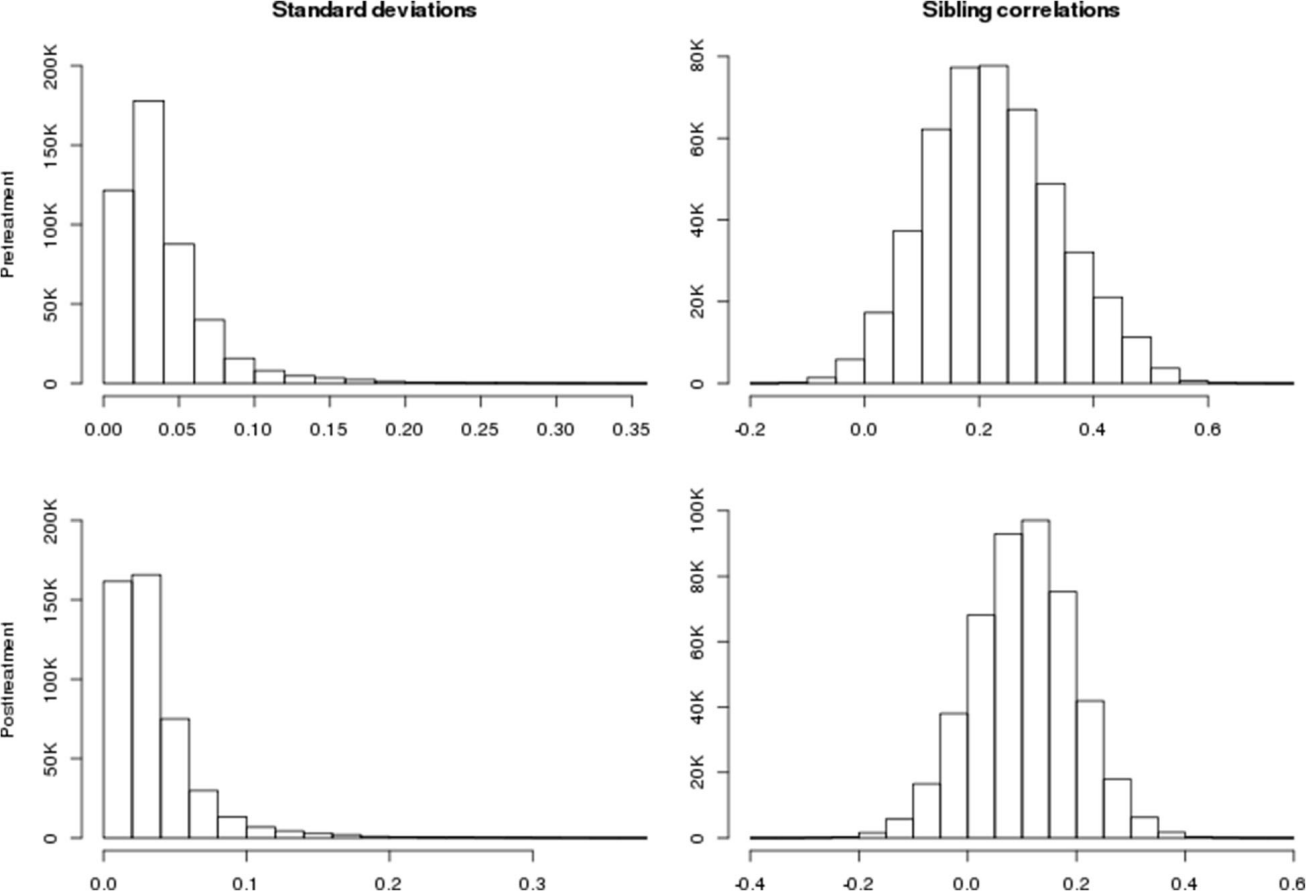
Distributions of methylation standard deviations and sibling correlations pre- and posttreatment with fenofibrate

**Fig. 2:**
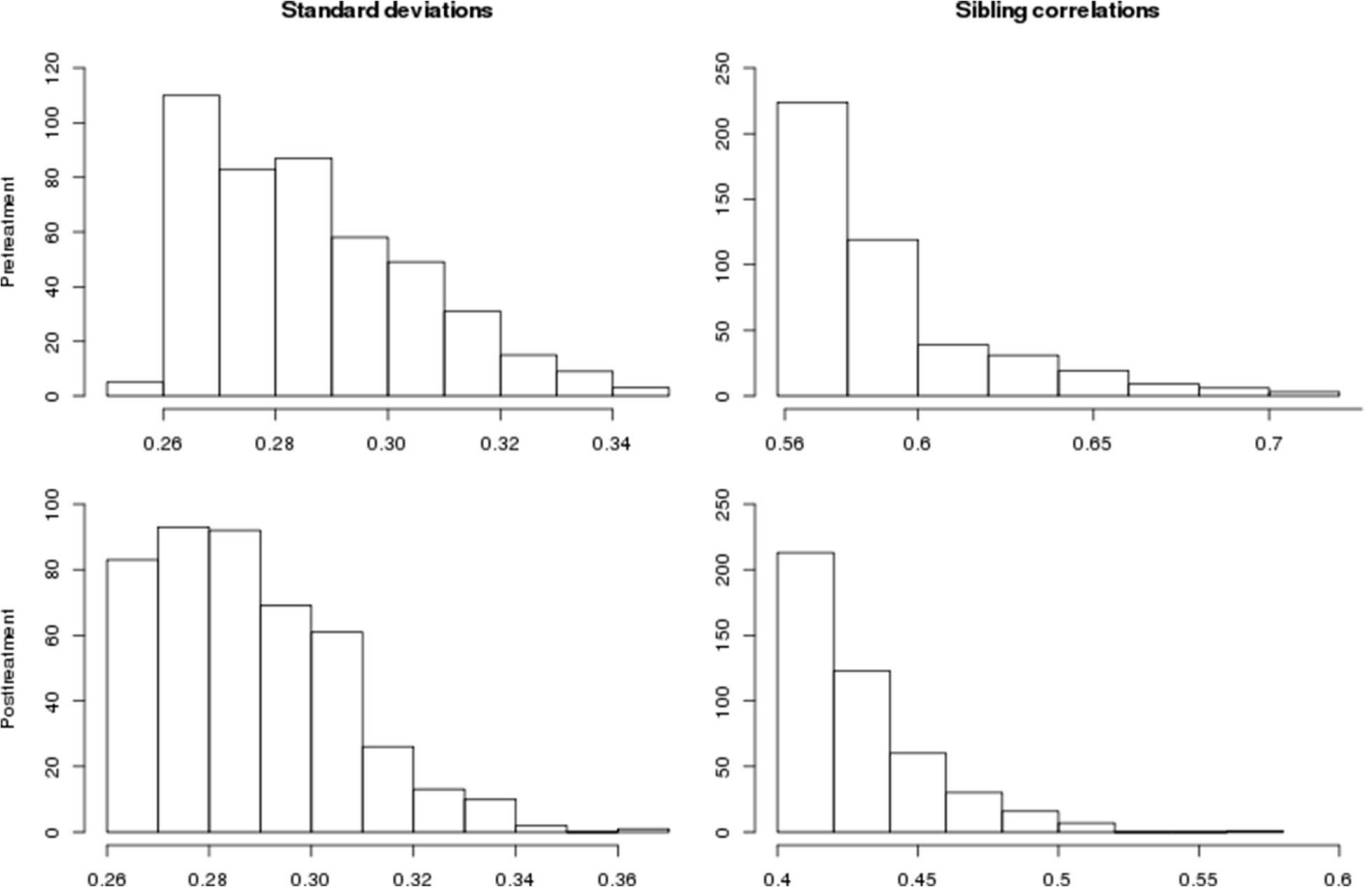
Outliers (top 0.1%) of distributions of methylation sibling correlations and standard deviations pre- and posttreatment with fenofibrate

Figure 1 presents the pre- and posttreatment SD and scor histograms. As Fig. 1 shows, even though these distributions are very similar, the ranks of the individual CpG sites within those distributions differ. Our analyses of target CpG sites with a genetic response to fenofibrate focus on the sites beyond the 0.1% (450 sites) CpG threshold in these distributions. Figure 2 shows their histograms. Twice the sibling correlation is an upper bound of the heritability of the methylation levels, indicating that these outlier CpG sites may have highly heritable methylation levels.

To identify CpG sites that are familial and variable we searched for shared outliers posttreatment and compared the results using the same analysis we used pretreatment. Although the pretreatment SD and scor distributions have no common outliers, the posttreatment scor and SD outliers shared 16 common sites (see Table 1), where the genes associated with the 16 sites are listed in alphabetical order, along with their chromosomes. Three sites listed at the bottom of the table do not have an associated gene. The fourth and fifth columns of Table 1 give the CpG ranks in the pre- and posttreatment SD distributions with the SD estimates in parentheses. The sixth and seventh columns give analogous information for scor. When comparing outliers pre- and posttreatment, 36 (8%) of SD and 449/450 (nearly 100%) of scor outliers differ.

**Table 1:** Associated genes and changes in SD and scorr for highlighted outlier CpG sites

CpG	Chr	Gene	Post SD rank (est^a^)	Pre SD rank (est)	Post scor rank (est)	Pre scor rank (est)
24,309,769	12	*A2ML1*	365 (0.27)	351 (0.27)	210 (0.42)	30,906 (0.41)
12,208,638	11	*ACTN3*	242 (0.28)	290 (0.28)	443 (0.4)	6697 (0.49)
**9307883**	**9**	***ANAPC2***	**404 (0.27)**	**505 (0.25)**	**314 (0.41)**	**110,943 (0.31)**
4,888,234	1	*FCRLA*	345 (0.27)	327 (0.27)	281 (0.42)	239,089 (0.21)
16,140,565	3	*FHIT*	47 (0.31)	95 (0.3)	169 (0.43)	70,600 (0.35)
1,778,345	1	*GDAP2*	182 (0.29)	318 (0.27)	391 (0.41)	36,074 (0.4)
3,796,003	16	*KCTD5*	218 (0.29)	245 (0.28)	180 (0.43)	176,434 (0.26)
**16,675,926**	**1**	***KIAA1804***	**435 (0.26)**	**613 (0.24)**	**395 (0.41)**	**231,790 (0.22)**
5,023,192	2	*NDUFA10*	38 (0.31)	32 (0.32)	206 (0.42)	153,842 (0.27)
17,040,924	11	*OR52M1*	63 (0.31)	132 (0.3)	37 (0.47)	35,002 (0.4)
8,210,706	14	*SERPINA5*	293 (0.28)	347 (0.27)	331 (0.41)	21,063 (0.43)
13,989,295	17	*SKA2*	108 (0.3)	105 (0.3)	426 (0.4)	38,937 (0.4)
10,890,644	10	*TUBAL3*	129 (0.3)	98 (0.3)	66 (0.45)	76,987 (0.34)
3,221,390	1		274 (0.28)	227 (0.28)	173 (0.43)	90,915 (0.33)
20,086,657	17		187 (0.29)	110 (0.3)	51 (0.46)	19,044 (0.44)
22,274,273	6		254 (0.28)	200 (0.29)	247 (0.42)	53,745 (0.37)

Sites in bold show a change in outlier status for both scor and SD

^a^*Est* refers to the estimate, rather than rank, of SD or scor in that sample

To illustrate the information in Table 1, in the first row, site 24,309,769 is on chromosome 12 and is associated with gene *A2ML1*. The SDs of methylation levels pre- and posttreatment are the same (.27) and the ranks in the pre- and posttreatment distributions have a marginal difference (365 and 351). Although the sibling correlations pre- and posttreatment (0.42 and 0.41) are almost identical for this site, their ranks differ substantially between the pre- (30,906) and posttreatment (210) distributions. Because the SD ranks put *A2ML1* in the outlier category pre- and posttreatment, we do not view this a providing strong support for a genetic response to the treatment with fenofibrate, even though the shift in rank in the scor distribution provide support for a genetic contribution to methylation levels. Although much of the table reflects a similar pattern, 2 sites and their corresponding genes are in bold because there is a change in outlier status for both scor and SD. We used the criterion that the pretreatment ranks for scor and SD do not meet our outlier definition. Two genes, *ANAPC2* and *KIAA1804*, meet this criterion, and became our strongest candidates for having a genetic response to fenofibrate.

**Fig. 3:**
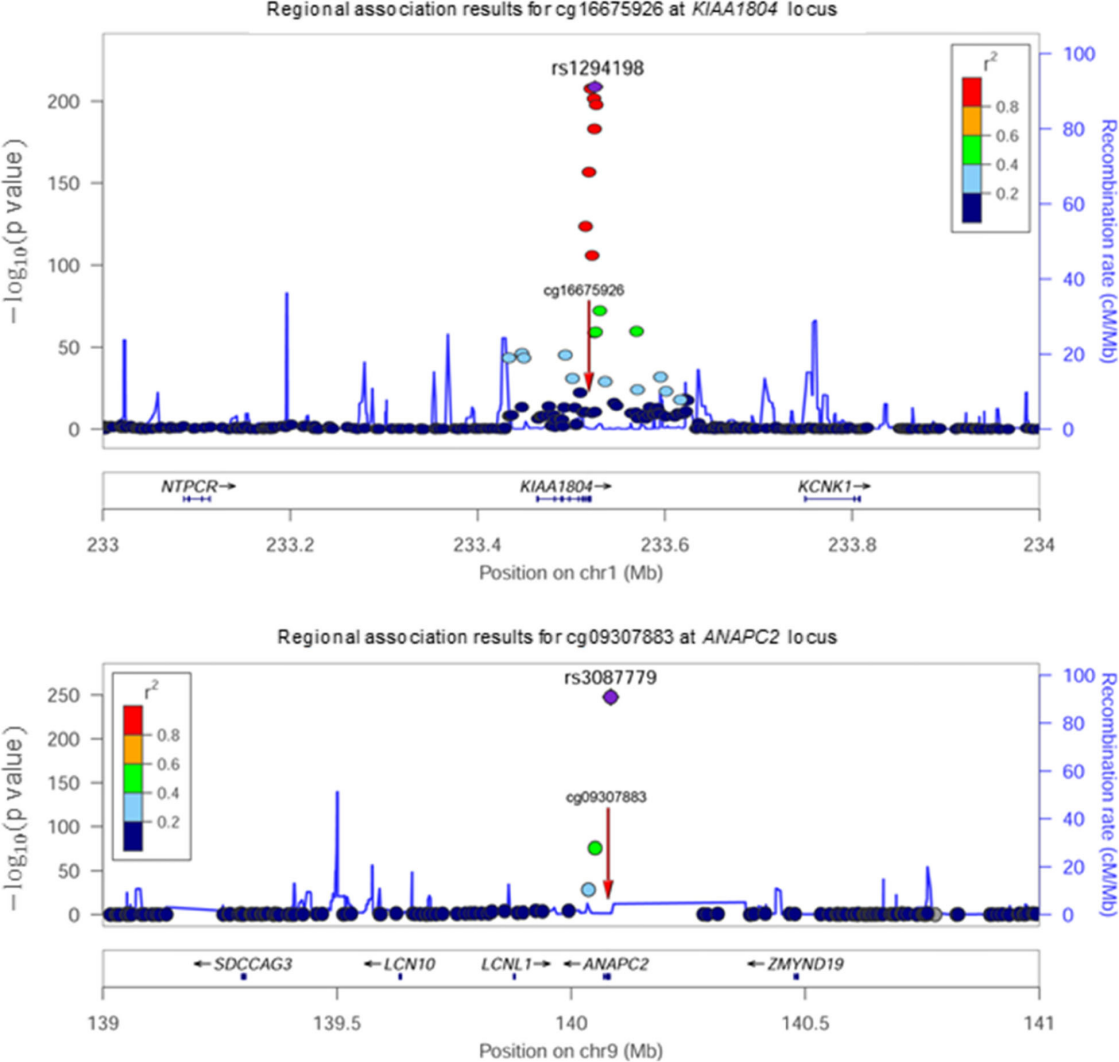
SNPs associated with methylation levels (me QTL) at fenofibrate-responsive candidate genes

We conducted meQTL analyses for *ANAPC2* and *KIAA1804* to identify genetic contributions to their methylation levels. For *KIAA1804*, there is a very strong GWAS peak with the lead SNP, rs1294198 having a *p* value <1e-200. For *ANAPC2*, there is also a strong GWAS peak (*p* < 3e-248), with the lead SNP rs3087779. Figure 3 presents the results of the SNP association analyses for *KIAA1804* and *ANAPC2*, with the methylation sites shown on the plots with red arrows. For both genes, linkage disequilibrium estimates between the lead SNP and the other associated SNPs are correlated with the sizes of their association signals. The SNP driving the association at *ANAPC2* is somewhat straightforward, and is likely to be the lead SNP. However, identifying the SNP (or SNPs) driving the association with methylation at *KIAA1804* is not straightforward.

The base pair range for *KIAA1804* is 233,463,514 to 233,520,894. The lead SNP, rs1294198, is to the right and downstream of the gene at 233,525,375, and the CpG, 16,675,926, is at 233,518,998, within the gene. The base pair range for *ANAPC2* is 140,069,236 to 140,083,057. The lead SNP, rs3087779, is to the right and upstream of the gene at 140,084,485, and the CpG, 09307883, is at 140,077,638, within the gene.

## Discussion

This study was designed to capitalize on the longitudinal nature of the GAW20 data, and assess whether there are CpG sites responsive to treatment with fenofibrate. To answer this question, our aim was to detect any such CpG sites. Initial analyses and discussions at GAW20, made us aware of a batch effect, which precluded testing the CpG sites for a fenofibrate response using a direct comparison in their values, a preferred method of analyzing pre- and posttreatment changes in methylation levels. This would be accomplished by pairing the pre- and posttreatment measures for an individual and using the difference or ratio of the methylation levels to identify those CpG sites that are significant. Given the confounding batch effect, we chose, instead, to employ an indirect approach targeting CpG sites exhibiting posttreatment genetic effects that were not seen pretreatment.

A number of study design choices arose. First, we chose to analyze the full pre- and posttreatment samples rather than the reduced sample of 446 overlapping individuals and 102 sibling pairs. To make that choice, we assumed that these full samples are unbiased representations of the pre- and posttreatment populations, and reasoned that the full samples provide greater power to estimate SD and scor. We also assumed that everyone in the posttreatment sample received the full treatment with fenofibrate, although individual treatment histories were not available. The reduced samples provide a single consistent, but not necessarily unbiased, sample and have lower power because our analyses do not capitalize directly on the paired aspect of these data. However, we also conducted the same analyses in the reduced sample, and, unfortunately, did not find any CpG sites meeting our criteria for being fenofibrate responsive. This led us to a second analytic choice regarding the threshold used for considering an observation to be an outlier.

In the larger pre- and posttreatment samples, we set an arbitrary 0.1% threshold for identifying outliers. If this had failed in the full sample, our plan was to set a more permissive threshold of 0.5%, and if that failed, set an even more permissive threshold of 1%. Using the 0.5% cutoff in the reduced sample, SD is ranked 463 posttreatment and 635 pretreatment, and scor is ranked 1000 posttreatment and 17,955 pretreatment; *KIAA1804* is selected again.

Additional design factors to consider are the criteria to detect evidence of a genetic effect on the CpG methylation levels. Those CpG sites with outlier SDs can be reflecting the effects of SNPs on their methylation levels. Although a single SNP can cause a distribution to become bimodal, the effects of multiple SNPs are better detected using SD. In addition, screening 450,000 CpG sites is a daunting task.

In summary, our analysis that uses outliers of the familiality and variability distributions of CpG methylation levels identified CpG sites exhibiting patterns consistent with a genetic influence on their response to a 3-week treatment with fenofibrate. The analysis is based on a molecular model that postulates that fenofibrate changes the activation levels of transcription factors that bind to sites harboring SNPs, and the SNPs introduce a methylation pattern that is consistent with the influence of the genetic variation on expression and ultimately methylation. Using an indirect approach capitalizing on this model, we identified 2 CpG sites, at *KIAA1804* and *ANAPC2,* which are consistent with a genetic influence*.* A search for genetic factors likely to contribute to their methylation levels identified their meQTL. At *KIAA1804*, the linkage disequilibrium illustrated in Fig. 1 precludes identifying the specific SNP(s) responsible for this genetic effect. For *ANAPC2*, because of the limited number of highly significant associations, the lead SNP, rs3087779, appears to be the one responsible. For both *KIAA1804* and *ANAPC2,* predictions of transcription factor binding sites for the 2 alleles of their lead SNPs show allele specific differences, providing support for our underlying model.

Although our approach to detect fenofibrate responsive CpG sites is indirect, we feel that it has been successful in identifying two CpG sites for future investigations.

## Conclusions

A genetic approach that uses the analysis of outliers of pre- and posttreatment familiality and variability distributions has been successful in identifying fenofibrate responsive CpG sites.
